# Cortical lamina technique: A therapeutic approach for lateral ridge augmentation using guided bone regeneration

**DOI:** 10.4317/jced.53008

**Published:** 2017-01-01

**Authors:** Shree-Lakshmi Deepika-Penmetsa, Raison Thomas, Tarun-Kumar Baron, Rucha Shah, Dhoom-Singh Mehta

**Affiliations:** 1 BDS, Post Graduate Resident, Department of Periodontics, Bapuji Dental College & Hospital, Davangere- 577004, Karnataka, India; 2 MDS, Professor, Department of Periodontics, Bapuji Dental College & Hospital, Davangere- 577004, Karnataka, India; 3 MDS, Professor, Department of Periodontics & Implantology, Bapuji Dental College & Hospital, Davangere- 577004, Karnataka, India; 4 MDS, Senior Lecturer, Department of Periodontics, Bapuji Dental College & Hospital, Davangere- 577004, Karnataka, India; 5 MDS, FICD, FADI, FISOI., Professor & Head, Department of Periodontics, Bapuji Dental College & Hospital, Davangere- 577004, Karnataka, India

## Abstract

**Background:**

The present study aimed at evaluating the efficacy of a novel technique, the bone lamina technique, in horizontal ridge augmentation clinically & radiographically using a combination of allogenic cortical shell, particulate xenograft and resorbable collagen membrane.

**Material and Methods:**

Localized horizontal ridge defects, in ten patients (6 male, 4 female), with bucco-palatal ridge width less than 5 mm were included in this study. Localised ridge augmentation was performed using bone lamina technique with mineralised allogenic shell of 1 mm thickness trimmed to the appropriate size using stereo-lithographic models and fixed to the recipient site with stainless steel micro-screws of 1 mm diameter. The space between the shell & host bone was filled with particulate xenograft followed by placement of collagen membrane and primary closure of the site. Clinical parameters including ridge width before & after flap reflection & radiographic (CBCT) ridge width measurements were recorded pre-operatively,and six months after the augmentation procedure. Results obtained were analysed statistically.

**Results:**

The mean clinical ridge width before flap reflection (BFR), after flap reflection (AFR) & radiographically was 3.7 ± 0.74 mm, 2 ± 0.70 mm & 1.77 ± 0.71 mm respectively at baseline which increased to 6.8 ± 0.95 mm, 5.15 ± 0.98 mm & 4.90 ± 0.90 mm with a mean gain in ridge width of 3.1 ± 0.63 mm (*p*< 0.005), 3.15 ± 0.63 mm (*p*<0.005) & 3.13 ± 0.70 mm (*p*< 0.005) respectively.

**Conclusions:**

The present study demonstrates that bone lamina technique can be effective means of horizontal ridge augmentation and the use of mineralized allograft in combination with xenograft and collagen membrane leads to good amount of bone regeneration for subsequent implant placement.

** Key words:**Dental implant, guided bone regeneration, horizontal ridge defect, ridge augmentation.

## Introduction

With increasing demands of the patient regarding aesthetics and function, the need for implant supported reconstructions has substantially increased (1). Extensive research is being done to improve the success of implant therapy. Numerous changes in the alveolus that follow tooth loss, frequently compromises dental implant placement in a prosthetically ideal position. The natural remodelling processes of alveolar socket begin immediately after extraction and may result in up to 50 % resorption of the alveolar ridge (AR) within 3 months (2). Therefore, augmentation of an insufficient bone volume is often indicated prior to or in conjunction with implant placement to attain predictable long-term functional and aesthetic treatment outcome (3).

Although socket grafting with some of the bone substitutes have been able to limit the resorption of post-extraction alveolar ridge up to a certain extent, some amount of bone loss occurs inevitably. The quality of the new tissue in the socket varies broadly (4). Also, clinicians may come across deficient ridges in cases of traumatic extraction, periodontally hopeless tooth etc. Such cases are indicated for ridge augmentation prior to implant placement (2).

A plethora of surgical techniques have been described in the last four decades regarding reconstruction of deficient alveolar bone, e.g., use of particulate grafts, block grafts, ridge splitting or ridge expansion, and distraction osteogenesis (5). The choice of augmentation procedure depends on the location & size of defect, clinician’s experience & choice, patient preferences, affordability & feasibility of the procedure. The studies that exist for alveolar ridge augmentation using guided bone regeneration (GBR) techniques seem to yield comparable and favourable results (6).

The clinician must make the appropriate selection of graft material and technique based on the size, shape, and dimensions of the defect and its location in the mouth (6). When the defect is primarily horizontal in nature (Seibert Class I) with ridge width < 3.5 mm, the choice of augmentation procedures includes onlay block grafting & GBR. Autogenous bone blocks of minimum thickness (1 mm) have been used in combination with particulate graft and collagen membrane for ridge augmentation procedures with good results (7). However, donor site morbidity, unpredictable resorption, limited quantities available, and the need to include additional surgical sites are drawbacks related to autografts that have intensified the search for suitable alternatives.

Allografts can be used in particulate form or as a block graft, either alone or in combination with autogenous, xenogeneic, or alloplastic materials. Recently, a pilot study successfully described the bone lamina technique to manage horizontal ridge defects. They utilised a porcine cortical bone shield along with collagen membrane to maintain space as well as proper contour of the alveolar ridge which resulted in predictable bone regeneration (8). However, on thorough literature search we did not come across any study on use of cortical bone allografts in combination with collagen membrane and particulate xenograft for augmentation of horizontal ridge defects.The use of this combination provides us with advantages of less patient morbidity, adequate maintenance of space and contour of the alveolar ridge.

Hence, the aim of the present study was to evaluate clinically and radiographically the efficacy of the use of allogenic cortical bone lamina along with collagen membrane & particulate xenograft in the augmentation of localized horizontal ridge defects.

## Material and Methods

Ten patients (six male, four female) for this study were selected from the out-patient Department of Periodontics & Implantology. Each patient enrolled in the study was given a detailed verbal & written description of the risks & benefits of the proposed treatment in their own language & a signed consent was obtained from them before commencement of the study. Ethical approval for the study was obtained from the Institutional Ethical Committee.

Patients in the age group of 18-45 years with good general health and oral hygiene were considered for the study. These patients with localised horizontal ridge defects measuring < 4 mm in ridge width at the alveolar crest either in maxilla or mandible were included in the study (9). Pregnant women, lactating mothers, patients on bisphosphonate therapy or other drugs which affect the bone metabolism, history of periodontitis & smoking habit were excluded from the study.

**Figure 1:**
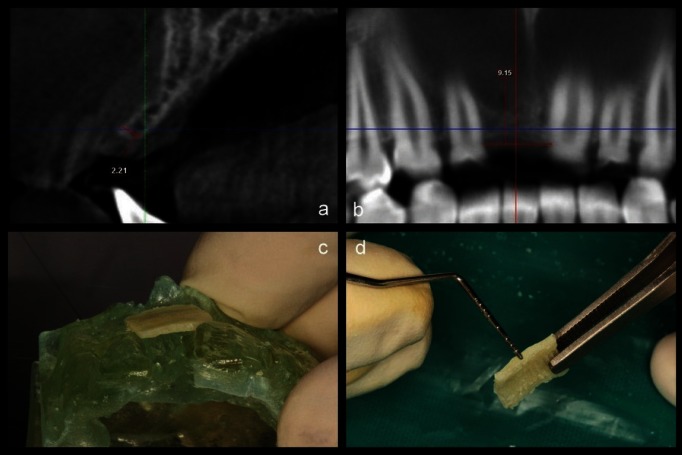
a) Pre-operative radiographic (saggital) view of ridge width. b) Pre-operative radiographic (panoramic) view of ridge width. c) 3-D stereolithographic model of the recipient site. d) Cortical lamina trimmed to appropriate dimensions.

On the first visit, complete medical and dental histories were recorded and thorough clinical examination was performed. Intra oral peri-apical radiograph of the edentulous site was assessed to exclude patients with vertical ridge defects. Routine blood investigations including complete hemogram, glycated haemoglobin (HbA1c) assay, rapid ELISA, HbSAg were performed to evaluate the general well-being of the patient. Oral hygiene instructions were given and oral prophylaxis was performed. In the recall visit 2 weeks after oral prophylaxis, the oral hygiene maintenance was assessed. A cone beam computed tomography (CBCT) scan of the region of interest was recorded. Diagnostic casts were prepared. Three dimensional stereo-lithographic models were obtained from the pre-recorded CBCT (Fig. 1a-c).

Pre-surgical protocol: Premedication included Augmentin 625 mg (Amoxicillin 500 mg + Clavulanic acid 125 mg) and Ketorol DT 10 mg (Ketorolac) was advised 1 hour prior to the surgery. Pre-procedural mouthrinse in the form of 0.2% Chlorhexidine gluconate mouthwash for 30 seconds was given. This was followed by disinfecting the extra-oral region of the face using 5% Povidone iodine. A cortical allograft shell of 1mm thickness (Tissue bank, Tata Memorial Hospital, Mumbai, India) was customised to the required dimensions based on stereolithographic model (Fig. 1d).

Surgical procedure: Adequate local anaesthesia was obtained at the surgical site using 2% lignocaine with of adrenaline (1:1,00,000 concentration). Mid-crestal incision using Bard Parker (BP) knife with 15c blade, was given which was extended as a sulcular incision on the surfaces of adjacent teeth. Vertical releasing incisions were given at the disto-buccal line angles of the adjacent teeth, connecting to the horizontal incision and extending few millimetres (mm) beyond muco-gingival junction. A full-thickness muco-periosteal flap was reflected buccally & palatal flap was reflected to just expose about 3 mm of bone to be able to stabilise the collagen membrane underneath the flap. After flap reflection, the recipient site was debrided & irrigated with sterile saline.

**Figure 2:**
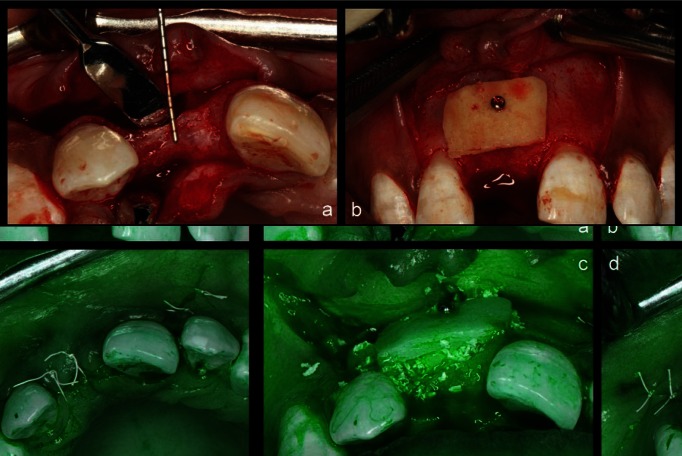
a) Clinical view of surgical site after flap reflection. b) Fixation of cortical shell to the recipient site with miniscrew. c) Particulate xenograft filled into the space between the cortical lamina & host bone. d) Primary closure of the site.

The clinical ridge width dimensions were measured before, and after flap reflection using UNC-15 periodontal probe (Hu-Friedy, USA). The ridge width was measured bucco-palatally in mid-buccal region at the level of alveolar crest (Fig. 2a). Radiographic measurements of bucco-palatal ridge width were recorded using CBCT scans taken pre-operatively. The ridge width was measu-red on sagittal section.

The customized allogenic shell with rounded margins was adapted to the recipient site and fixed in place with 1 or 2 stainless steel mini-screws of 1 mm diameter (Helmut Zeph, Germany) (Fig. 2b). The space between the shell and the recipient site was filled with particulate xenograft 0.5 to 1 mm diameter (Cerabone, Botiss, Germany) (Fig. 2c) followed by the placement of resorbable collagen membrane (Conform, Novabone, USA). The membrane was stabilised by tucking it underneath the palatal flap. The flaps were approximated to attain tension free primary closure with a combination of horizontal mattress and interrupted sutures (Fig. 2d). The vertical incisions were approximated using interrupted sutures (5-0 f-PTFE, Ethilon, USA).

Post-operative care:Written post-operative instructions were given and explained to the patient. Ice pack was advised for the first twelve hours and patients were asked to report in case of prolonged bleeding. Oral hygiene instructions were reinforced and antibiotics Augmentin 625 mg (Amoxicillin 500 mg + Clavulanic acid 125 mg thrice a day) and analgesics Maxrel (Diclofenac 50mg + Paracetamol 500mg thrice a day) were prescribed for 5 days. Chlorhexidine 0.2% mouthrinse was also prescribed to be used twice daily for 2 weeks. Patients were recalled on third day post-surgery for re-evaluation. At 14 days post-surgery, suture removal was performed followed by temporization in the same visit using acrylic teeth splinted to the adjacent teeth using stainless steel wire and composite. Following this the patient was kept under regular follow-up once a month for 6 months.

**Figure 3:**
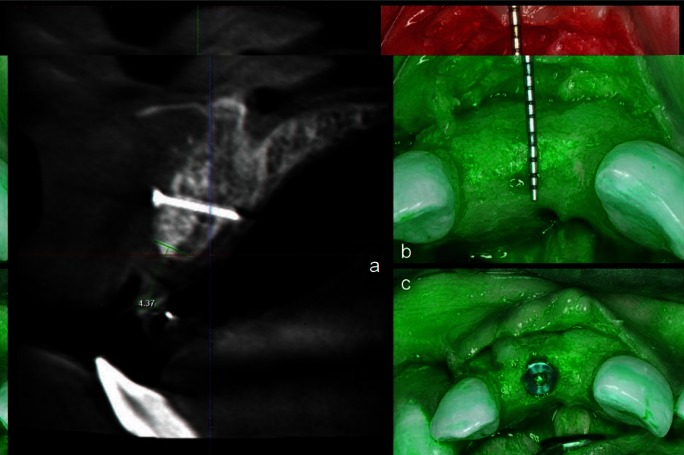
a) Six months post-operative radiographic (sagittal) view of augmented site. b) Clinical view of surgical site at re-entry after 6 months. c) Clinical view after implant placement.

At 6 months following augmentation procedure, CBCT was taken and measurements were made on the same sagittal section as that of pre-operative CBCT (Fig. 3a). This was ensured by taking the adjacent teeth as reference and keeping the distance from the adjacent teeth to the site being measured constant. The ridge width was obtained by measuring at the alveolar crest on sagittal section and the difference in ridge width was recorded.

All the values recorded were entered in a pro-forma & subjected to statistical analysis. Statistical analysis was done using SPSS software (Version 17.0). Wilcoxon signed rank test was used to analyse the changes in ridge width clinically and radiographically. Repeated measures ANOVA test was used to compare the mean values at different time intervals. For this study, *p* value of < 0.05 was considered to be statistically significant.

## Results

All the patients completed the follow up & there were no drop-outs. Post-operative healing of the surgical site was uneventful except for one patient where membrane exposure was seen 3 days after the procedure. This was managed by betadine (5 % Povidone iodine) irrigation of the site on 3rd, 7th & 14th day post-surgery, antibiotic therapy (Augmentin 625mg thrice a day for 5 days) and 0.2% Chlorhexidine rinses twice daily which led to satisfactory healing of the site.

At baseline, the mean clinical ridge width before flap reflection (BFR) was 3.7 ± 0.74 mm. At 6 months, it increased to 6.8 ± 0.95 mm with a mean difference of 3.1 ± 0.63 mm and the values obtained were statistically highly significant (*p*< 0.005). At baseline, the mean horizontal ridge width after flap reflection (AFR) was 2 ± 0.70 mm which increased to 5.15 ± 0.98mm six months after the augmentation procedure. A mean gain in ridge width of 3.15 ± 0.63 mm was observed and this difference was found to be statistically highly significant (*p*< 0.005). The mean radiographic ridge width was 1.77 ± 0.71 mm prior to augmentation, which increased to 4.90 ± 0.90 at six months following the surgical procedure which was found to be statistically highly significant with a mean gain of 3.13 ± 0.70mm (*p*< 0.005).

Following 6 months, surgical re-entry of the was done for implant placement (Fig. 3b,c) in augmented sites with sufficient ridge width, with average implant dimensions of 3.5 X 10.5 mm in all the cases. Details of each case are presented in  Table 1. All the implants were placed by single operator. Based on the tactile sensitivity, the operator assessed the regenerated bone to be normal to dense in all the cases (10). During implant insertion a torque value of ≥ 35 Ncm was obtained in all the cases suggesting good quality of the bone regenerated.

**Table 1:**
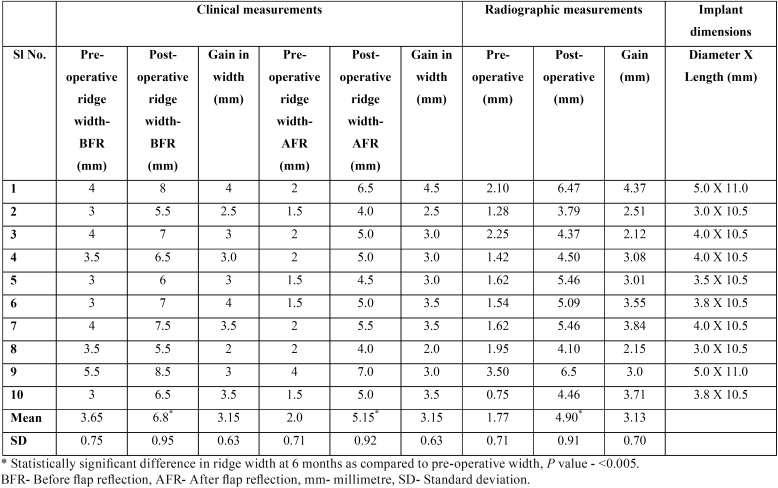
Clinical & radiographic measurements of ridge width at baseline & 6 months post-operatively.

## Discussion

Clinicians are routinely faced with the need to restore a single tooth in an otherwise non-restored dentition because of traumatic incidents, caries and congenitally missing teeth. In these situations, the treatment options include a traditional fixed partial denture, a resin-bonded restoration and an implant supported prosthesis (11). Implants offer significant advantages over resin-bonded or conventional bridges. They prevent the needless restoration of sound teeth adjacent to the edentulous area as would be required for a fixed partial denture. In instances where the adjacent teeth have no restorations, a single-tooth implant provides the opportunity to preserve the integrity of the existing teeth (11).

Advances in technology have altered our treatment philosophy in the replacement of a single tooth. Over the years, several concepts have emerged to obtain good osseo-integration and long-term stability of implants. The notion of placing implants in the sites with sufficient bone has changed to prosthetically driven implant placement (12). This includes pre-surgical implant site development, followed by implant placement & final prosthesis. Tooth loss inevitably leads to hard and soft tissue loss. Most of the remodelling of alveolar socket occurs in the first six months following extraction. In a systematic review by Tan *et al.*, it has been stated that horizontal dimensional reduction (3.79 ± 0.23 mm) was more than vertical reduction (1.24 ± 0.11 mm on buccal, 0.84 ± 0.62 mm on mesial and 0.80 ± 0.71 mm on distal sites) at 6 months (13). Thus horizontal ridge defects can be considered the most severe ones which require management before placement of implants.

This necessitates hard &/or soft tissue augmentation procedures for successful rehabilitation of the ridges with implant supported prosthesis. As implants placed in augmented sites have shown similar success rate to those placed in pristine bone (14), several techniques have been proposed for ridge augmentation. Guided bone regeneration, block bone grafting and ridge-split technique are commonly used for the management of horizontal ridge defects (15). Though several techniques have been proposed for the management of ridge defects, every procedure comes with its own limitations. Hence, search is still on to bring about techniques with lower number of limitations and high predictability.

Previously, in guided bone regeneration procedures block grafts alone or in combination with particulate graft, resorbable membranes or titanium mesh have been used to achieve the basic “PASS” principle for the success of the procedure. In the present case series, we have successfully used a thin allogenic cortical shell along with particulate xenograft, collagen membrane and miniscrews. Space maintenance is one of the basic principles for the success of GBR without which regeneration of bone is questionable (16). Collagen membranes have several desirable properties and have yielded favourable results with GBR (17). However, the major disadvantage of bio-resorbable membranes is the collapse of membrane and inability to maintain space in areas other than self-contained defects. To overcome this, allogenic cortical shell & slow resorbing particulate xenogenic graft materials have been used under the bio-resorbable membrane. Allografts are osteoconductive in nature. They gradually resorb and are replaced by host bone. It is hypothesized that the cortical plate of allogenic block graft will provide rigidity for fixation and also prevent any resorption during the healing phase (18).

The space remaining between the cortical bone lamina and the host bone was filled with particulate xenograft. Xenografts seem to be biocompatible, demonstrate osteoconductive properties, and undergo remodeling during a slow process. In studies where particulate xenograft was used, the particles were found to be well integrated in the regenerated bone (19).

In the present study,stereo-lithographic models have been procured from the pre-operative CBCT scan which acts as a surgical guide to plan the surgery & estimate the graft dimensions required to customize the block graft prior to surgery. This greatly reduces the chair-side time of the surgical procedure.

Similar combination technique done by Wachtel *et al.*, using porcine cortical shield and collagen membrane, showed that the resorption of cortical shield and collagen membrane occurred within 6 months (20). In the present study, re-entry was done 6 months after the augmentation procedure for implant placement. Post-operative healing of the surgical site was uneventful except for one patient where membrane exposure was seen 3 days after the procedure. This was managed by betadine irrigation of the site and antibiotic therapy (Augmentin 625 mg tid for 5 days) which led to satisfactory healing of the site.

The mean increase in ridge width clinically at the end of 6 months was 3.1 ± 0.62 (BFR) and 3.2 ± 0.63 mm (AFR). The mean post-operative bone width at 6 months was 5.15 ± 0.92 mm and the change in ridge width was statistically significant (*p* < 0.05). The mean pre-operative radiographic ridge width was 1.77 ± 0.71 mm which increased to 4.90 ± 0.91 mm at the end of 6 months. This mean gain in ridge width of 3.13 ± 0.70 mm was found to be statistically significant (*p* < 0.05).

Results obtained were comparable to other studies where similar combination of graft materials was used. In a study done by Geurs *et al.*, 72 sites with a mean pre-operative ridge width of 2.4 mm at the crest were treated with a combination of demineralized bone matrix, cortical cancellous chips and biodegradable synthetic barrier membrane. A mean gain in alveolar ridge width at the alveolar crest was 2.8 mm (21). von Arx *et al.* conducted a study where horizontal ridge augmentation with guided bone regeneration technique was done using a combination of autogenous block graft, particulate xenograft and collagen membrane. A mean gain in ridge width of 4.6 mm was found (22). As the results of the present study are comparable to that of gold standard autogenous block grafting, this technique can be considered effective in the management of horizontal ridge defects.

Implants placed in augmented sites are said to have similar survival rate as that placed in natural bone. In the present study, sufficient bone width was achieved following ridge augmentation and thus an implant of average diameter 3.5 to 4.5 mm and 10 mm in length was placed in each site, at the end of 6 months. Further long-term follow-up of these cases is required to determine the success of implants placed and also the augmentation procedure.

## Conclusions

Within the limitations of the present study, it can be concluded that the bone lamina technique using an allogenic cortical shell in combination with xenograft and collagen membrane can be used as an effective technique to manage the cases of horizontal ridge defects. The key to managing such defects is proper case selection. Further long term studies with larger sample size and long-term follow-up are necessary to substantiate the results obtained.
